# Intercostal Catheters Reduce Long-Term Pain and Postoperative Opioid Consumption after VATS

**DOI:** 10.3390/jcm13102842

**Published:** 2024-05-11

**Authors:** Marie-Christin Neuschmid, Florian Ponholzer, Caecilia Ng, Herbert Maier, Hannes Dejaco, Paolo Lucciarini, Stefan Schneeberger, Florian Augustin

**Affiliations:** 1 Department of Visceral, Transplant and Thoracic Surgery, Center of Operative Medicine, Medical University of Innsbruck, 6020 Innsbruck, Austriaflorian.ponholzer@i-med.ac.at (F.P.);; 2 Department of Anaesthesiology and Critical Care, Medical University of Innsbruck, 6020 Innsbruck, Austria

**Keywords:** VATS, intercostal catheter, pain management, opioid, postoperative pain

## Abstract

**Background/Objectives**: Pain after video-assisted thoracoscopic surgery (VATS) leads to impaired postoperative recovery, possible side effects of opioid usage, and higher rates of chronic post-surgery pain (CPSP). Nevertheless, guidelines on perioperative pain management for VATS patients are lacking. The aim of this study was to analyze the effectiveness of intercostal catheters in combination with a single shot intraoperative intercostal nerve block (SSINB) in comparison to SSINB alone with respect to opioid consumption and CPSP. **Methods**: Patients receiving an anatomic VATS resection between 2019 and 2022 for primary lung cancer were retrospectively analyzed. A total of 75 consecutive patients receiving an ICC and SSINB and 75 consecutive patients receiving only SSINB were included in our database. After enforcing the exclusion criteria (insufficient documentation, external follow-ups, or patients receiving opioids on a fixed schedule; *n* = 9) 141 patients remained for further analysis. **Results**: The ICC and No ICC cohort were comparable in age, gender distribution, tumor location and hospital stay. Patients in the ICC cohort showed significantly less opioid usage regarding the extent (4.48 ± 6.69 SD vs. 7.23 ± 7.55 SD mg, *p* = 0.023), duration (0.76 ± 0.97 SD vs. 1.26 ± 1.33 SD days, *p* = 0.012) and frequency (0.90 ± 1.34 SD vs. 1.45 ± 1.51 SD times, *p* = 0.023) in comparison to the No ICC group. During the first nine months of oncological follow-up assessments, no statistical difference was found in the rate of patients experiencing postoperative pain, although a trend towards less pain in the ICC cohort was found. One year after surgery, the ICC cohort expressed significantly less often pain (1.5 vs. 10.8%, *p* = 0.035). **Conclusions**: Placement of an ICC provides VATS patients with improved postoperative pain relief resulting in a reduced frequency of required opioid administration, less days with opioids, and a reduced total amount of opioids consumed. Furthermore, ICC patients have significantly lower rates of CPSP one year after surgery.

## 1. Introduction

Lung cancer is still the most common cause of cancer-related deaths in the western world [1]. Due to improvements in diagnosis over the last decades and, therefore, increased diagnoses in early stages, surgical treatment will become more frequent [2,3]. In terms of surgical approaches, minimally invasive video-assisted thoracoscopic surgery (VATS) gradually replaced open thoracotomy within the last two decades. The distinct advantages of a VATS approach primarily lies in its reduced invasiveness, as well as the associated decrease in postoperative pain [4,5]. Yet, a significant number of patients suffer from severe pain or even chronic pain after surgery [6,7,8].

Approximately 86% of all patients who undergo any kind of surgery experience postoperative pain [9]. About 4% of all patients suffer from severe pain (NRS = 7–10) on the first day after VATS surgery and up to 35% develop some chronic post-surgery pain (CPSP) [10]. The surgery-related components, for example, are the duration of surgery, number of incisions, or time until drainage removal. A considerable part of chronic pain syndromes after VATS surgery is a result of inadequate pain control in the early postoperative phase [5,6].

Currently, very heterogenous concepts are used in postoperative pain management. Thoracic epidural analgesia (TEA) and single shot intercostal nerve block (SSINB) represent the current majority of techniques used for pain control [7]. Even though TEA is a very effective method, several disadvantages may arise, such as immobilization, bladder dysfunction, and phases of hypotension [11,12].

SSINB provides the same efficiency as TEA and is associated with less side effects. The issue of this method is the duration of the pain control, which is only for a limited period of time because of the single shot application [11,12].

An advantageous method would be the use of intercostal catheters (ICC). [7] ICCs offer several benefits, such as targeted pain relief, the reduction of systemic side effects of opioid usage like nausea, dizziness, or respiratory depression, the possibility of continuous pain control, and, therefore, the opportunity for early mobilization. Additionally, the risks for pneumonia and thrombosis are reduced by the fact that patients with ICC are more likely to take part in physical and respiratory therapy and can be discharged more promptly. Furthermore, the operating time is not prolonged by the insertion of an ICC during VATS lobectomy or segmentectomy [7,13,14].

Further research and clinical studies are warranted to explore the potential benefits of the ICC method in optimizing postoperative pain management and reducing opioid-related complications [15,16,17,18].

Our group [7] was able to present positive effects of ICC placement in terms of reduced opioid usage in an analysis of the first patients after ICC introduction. Nonetheless, there are almost no data considering the effects on chronic pain and with larger patient cohorts.

This analysis aims to explore the possible advantages of ICC use for postoperative pain management in a larger patient cohort and with a longer follow-up to cover differences in CPSP.

## 2. Materials and Methods

### 2.1. Patient Selection

Patients who underwent anatomical VATS resection between 2019 and 2022 as treatment for primary lung cancer at our institution were included in this analysis. ICCs were implemented at our department in September 2019. The study was conducted in accordance with the Declaration of Helsinki (as revised in 2013). Permission was granted by the local ethics committee.

Patients with no comprehensive documentation of the postoperative opioid consumption, or postoperative pain assessments, or with external follow-ups were excluded. A total of 75 consecutive patients receiving an ICC and SSINB and 75 consecutive patients receiving only SSINB were included in our database. In the next step, patients receiving opioids on a fixed schedule were also excluded from analysis to avoid possible confounders (*n* = 9). Main reasons for a fixed schedule was catheter dislodgement or malfunction. After enforcing the exclusion criteria, 141 patients remained for further analysis.

### 2.2. Data Collection

Collected data included age, sex, type of operation, length of operation, placement of an ICC, duration until chest drain removal, postoperative opioid usage, postoperative complications, comorbidities, and pain assessments during oncological follow-up.

### 2.3. Study Endpoints

Assessment of pain was accomplished by asking patients if they experience pain related to thoracic surgery (e.g., pain at the incision site, pain during coughing, etc.). Pain was rated on a numeric rating scale (NRS) from 0 to 10. Follow-up visits in the outpatient clinic were scheduled 2 weeks, and 3, 6, 9, and 12 months after surgery. Primary endpoints of this study were postoperative pain after discharge from the hospital, as well as opioid usage and its duration during the initial stay at our department.

### 2.4. Surgical Technique and Catheter Placement

As described earlier [7], surgery was performed in a standardized manner with an anterior three-port VATS approach using the Copenhagen technique. It consists of a 2.5–3 cm-long anterior incision between the nipple and lower point of the scapula in the fourth or fifth intercostal space and two 1 cm-long incisions in the seventh and eighth intercostal space. The smaller anterior port is then used for the camera [19]. At the end of surgery, a single chest drain is inserted through the camera incision.

Independent of catheter placement, every patient received a SSINB with bupivacaine 2.5 mg/mL, which was injected in the intercostal spaces III–IX. Catheter placement into the subpleural space was performed with a 16G Tuohy needle at the end of surgery, in the same intercostal space as the thoracic drain. Starting in the postoperative care unit, 2 mg/mL of ropivacaine was continuously injected with a preset flow rate of 6 mL/h, administered by the same pump system as used for epidural anesthesia.

### 2.5. Peri- and Postoperative Pain Management

Patient’s conditions and comorbidities determined whether general anesthesia was performed with either propofol and remifentanil or sevoflurane plus remifentanil.Postoperative pain management was standardized with intravenous administration of one gram of Paracetamol and one gram of Metamizol three times per day on a fixed schedule for the first two postoperative days (POD). On POD 2, Paracetamol and Metamizol was oralized. Additionally, intravenous opioids were prescribed as rescue medication for severe peaks of pain, categorized as NRS > 5. For data analysis and calculations, the morphine equivalents of the prescribed opioids were used.

ICCs were removed at the day of chest drain removal or on POD 3, depending on which occurred earlier.

### 2.6. Pain Scoring and Documentation

We achieved a quantification by using the numerical rating scale (NRS) marked from 0 (no pain) to 10 (worst pain imaginable): mild pain is ranked 1–4, moderate pain 5–6, and 7–10 specifies severe pain [20,21,22].

Pain scores were assessed three times daily. A reassessment of pain levels was documented during postoperative consultations, which took place two weeks after discharge from the hospital, and then three, six, nine, and twelve months later.

### 2.7. Statistical Analysis

Student’s *t*-test was used for comparison of means, as the t-test was proven to show robustness even if the assumption of normality is violated [23,24,25]. Categorical variables were compared with Fisher’s exact test. Medians were evaluated by using K-sample test. Results with *p*-values < 0.05 were interpreted as statistically significant. Statistical analysis was performed using IBM SPSS Statistics 29 (IBM Corp., Armonk, NY, USA).

## 3. Results

### 3.1. Patient Demographics

The median age was 65.00 years (mean: 64.47). Seventy-five (53.2%) patients were female and sixty-six (46.8%) male. Of all patients, 67 (47.52%) received an ICC and SSINB, while 74 (52.48%) patients only received SSINB. The median age of patients with ICC was 65.00 years (mean: 64.23 years) and 65.00 years for the No ICC cohort (mean: 64.73 years) (*p* = 0.766). The median length of stay was 6.00 days for both groups (*p* = 0.536). The rate of left side resections was lower in both groups with 20 (29.9%) in the ICC and 29 (39.2%) in the No ICC cohort. The distribution of lobes was also balanced in both cohorts (*p* = 0.909), with 28 (41.8%) vs. 28 (37.8%) lower lobe, 31 (46.3%) vs. 39 (52.7%) upper lobe, 7 (10.4%) vs. 6 (8.1%) middle lobe, and 1 bilobectomy in each group. The frequency of diabetes mellitus (DM) was also analyzed to avoid possible distortions due to this comorbidity. Seven (10.4%) patients in the ICC cohort suffered from DM and eleven (14.9%) in the No ICC cohort. The demographics are shown in Table 1.

The duration of the operation did not differ between cohorts (ICC: 153.16 vs. No ICC: 155.68 min, *p* = 0.681).

### 3.2. Extent of Opioid Usage

**Figure 1:**
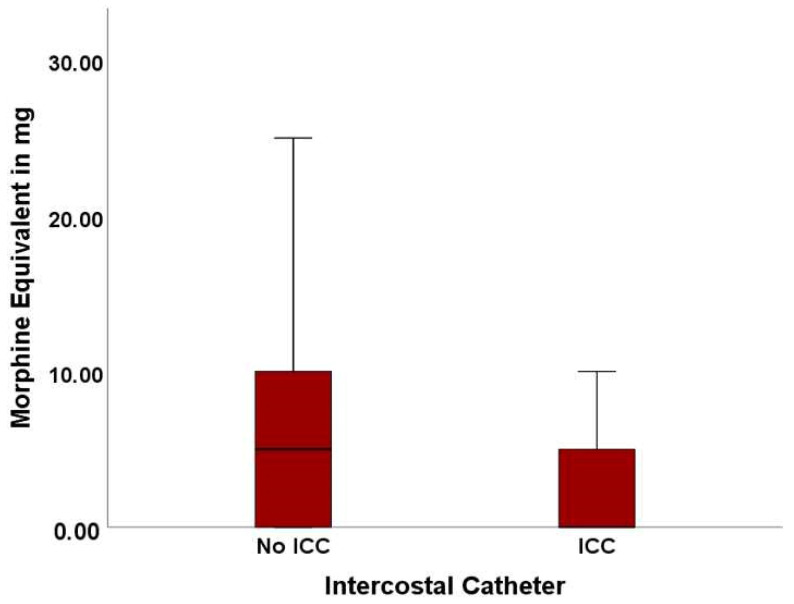
Opioid usage in the No ICC and ICC cohort.

One endpoint of this study was the usage of opioids during the complete time of hospitalization. With an average of 4.48 ± 6.69 SD mg of morphine equivalent, significantly lower doses of opioids were found in the ICC cohort compared to an average of 7.23 ± 7.55 SD mg in the No ICC cohort (*p* = 0.023). The consumed morphine equivalent is visualized in Figure 1.

Since the removal of the ICC is on POD 3 or contemporaneous with chest drain removal, the data were analyzed for these first three days after surgery separately. During POD 0–3, patients in the No ICC cohort showed a significantly higher opioid consumption with a mean of 6.49 ± 6.61 SD mg morphine equivalent in comparison to 4.10 ± 5.83 SD mg in the ICC cohort (*p* = 0.025).

**Figure 2:**
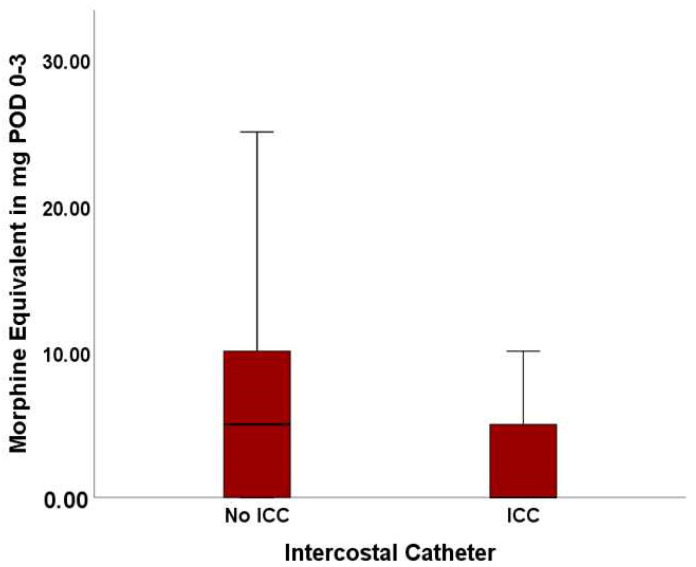
Opioid usage during POD 1–3 in the No ICC and ICC cohort.

The consumed morphine equivalent during POD 0–3 is visualized in Figure 2.

### 3.3. Duration of Opioid Usage

**Figure 3:**
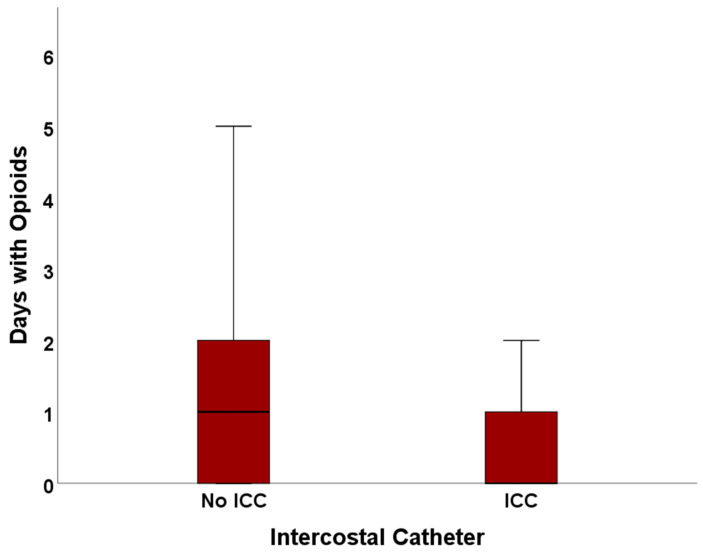
Duration of opioid usage in the No ICC and ICC cohort.

In the ICC cohort, patients required opioids for significantly fewer days with a mean of 0.76 ± 0.97 SD days in comparison to 1.26 ± 1.33 SD days in the No ICC cohort (*p* = 0.012), as shown in Figure 3.

More than half (50.7%) of all patients with ICC did not need opioids at all. In the No ICC cohort, only 36.5% had zero days of opioid usage. Although this difference seems remarkable, no statistical significance was found (*p* = 0.093). In addition, 40.5% of patients in the No ICC cohort required opioids for two or more days in comparison to 17.9% in the ICC cohort (*p* = 0.005).

### 3.4. Frequency of Opioid Usage

We analyzed the rate of how often patients asked for opioids as rescue medication.

Over the total length of hospitalization, patients with ICC asked 0.90 ± 1.34 SD times for further pain medication, compared to 1.45 ± 1.51 SD times in the No ICC cohort (*p* = 0.023).

When only analyzing data until POD 3, patients with ICC asked, on average, 0.82 ± 1.17 SD times for opioid rescue medication vs. 1.30 ± 1.32 SD times in the No ICC cohort (*p* = 0.025).

**Figure 4:**
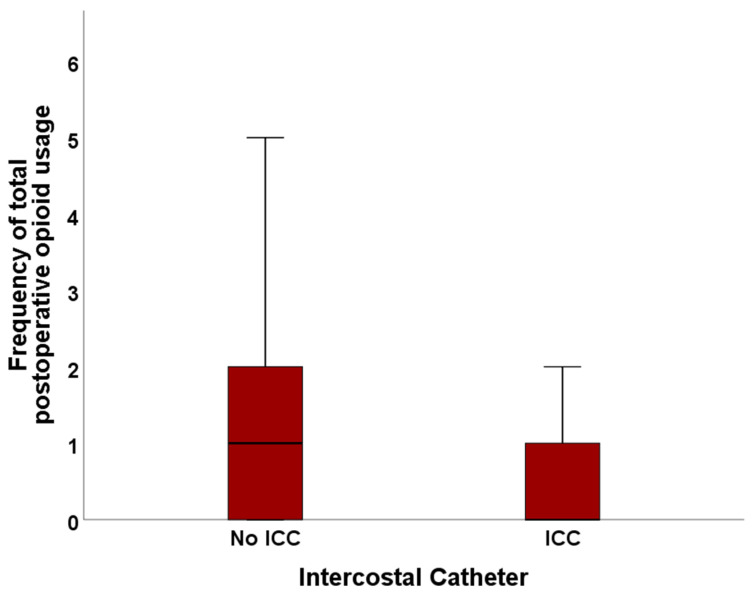
Frequency of opioid usage in the No ICC and ICC cohort.

**Figure 5:**
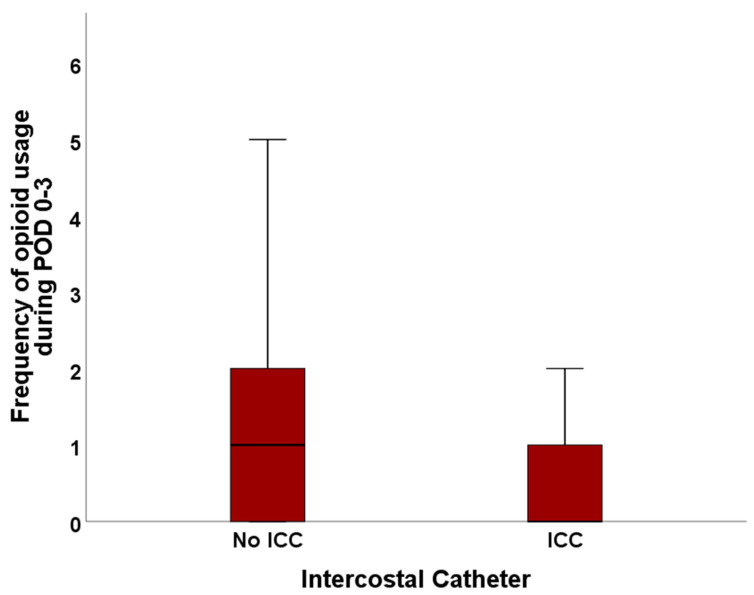
Frequency of opioid usage for the first three postoperative days in the No ICC and ICC cohort.

The median number of requests for the escalation of pain medication was 0.00 for ICC patients and 1.00 for patients with no ICC (total length of stay: *p* = 0.007; first three post-surgical days: *p* = 0.006). The frequency of opioid usage during POD 0–3 and the total stay after surgery is visualized in Figure 4 and Figure 5.

### 3.5. Pain during Oncological Follow-Up

Although a higher number of patients with pain is seen in every follow-up during the first nine months in the No ICC cohort, no statistical significance was found. In the first follow-up assessment, 34.3% of the people with ICC and 41.9% of patients without ICC complained about pain (*p* = 0.389); after three months, it was 11.9% compared to 21.6% (*p* = 0.178); after six months, 7.5% vs. 16.2% (*p* = 0.127); and, nine months after surgery, 6% vs. 9.5% (*p* = 0.538).

**Figure 6:**
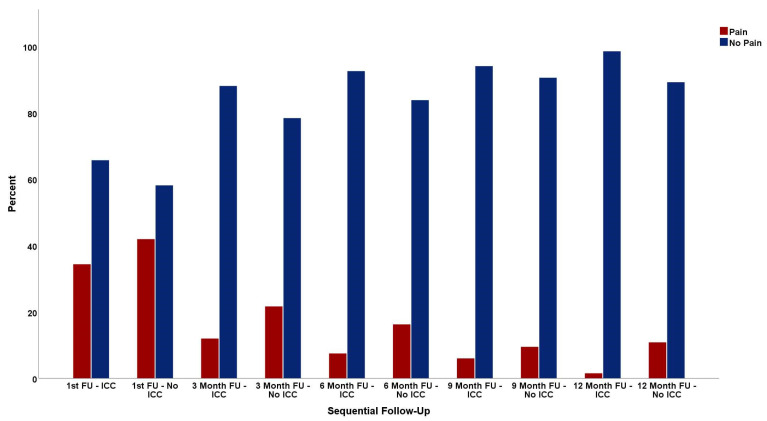
Pain in follow-up assessments.

Nevertheless, in the one-year follow-up, patients from the ICC cohort experienced less pain. Furthermore, 10.8% of all patients, who initially had no ICC placed, still suffered from pain 12 months after the operation compared to only 1.5% of patients with ICC treatment (*p* = 0.035), as visualized in Figure 6.

## 4. Discussion

In this follow-up study, we succeeded in confirming the results from our previously published pilot study [7]. We were able to show the superiority of ICC in combination with SSINB compared to SSINB alone in terms of opioid use. These data may be used to minimize opioid usage to reduce the possible associated downsides, while, at the same time, providing sufficient pain control.

The analysis showed statistically significant advantages for the ICC group regarding the amount, duration, and frequency of opioid usage after anatomical VATS resections. With this study, we were able to strengthen the hypothesis of ICCs, reducing the amount of required opioids for pain management with an average use of 4.48 mg of morphine equivalent for the complete time of hospitalization and 4.10 mg in the first three days after surgery for the ICC cohort. This is in contrast to 7.23 mg of morphine equivalent for the complete length of stay and 6.49 mg for the first three postoperative days in the No ICC cohort, providing a remarkable reduction of opioids (Total Length of Stay: *p* = 0.023; Postoperative Days 0 to 3: *p* = 0.025). Furthermore, the ICC group showed significantly less days without opioid use at all. Only 33 patients (49.3%) had opioids administered in comparison to 46 (62.2%) patients without an ICC (*p* = 0.012).

Requests of pain medication, or peak pain, also need to be optimized to reduce opioid consumption. We found significantly less requests for opioid rescue medication in the first three postoperative days in the ICC cohort, with 0.82 requests compared to 1.30 times in the No ICC cohort (*p* = 0.025), further proving the effectivity of ICCs for constant and reliable pain management. This difference could also be seen for the complete length of stay (0.90 vs. 1.45 requests, *p* = 0.023).

Not only was the frequency, amount, and duration of opioid administration positively influenced by ICC, but also the long-term pain control and possible subsequent development of CPSP. In every follow-up consultation, which took place two weeks after dismission from the hospital, and then three, six, nine, and twelve months after surgery, more patients with pain occurred in the No ICC cohort (first follow-up: *p* = 0.389; 3-month follow-up: *p* = 0.178; 6-month follow-up: *p* = 0.127; and 9-month follow-up: 0.538). Although statistical relevance was only found for the 12-month follow-up (*p* = 0.035), one can already see a trend towards better pain control in the first month after surgery as well. With 8 (10.8%) patients of the No ICC group, who still suffered from pain, vs. 1 (1.5%) patient of the ICC group, ICCs may provide a valuable tool with which to reduce the occurrence of CPSP.

The amount of CPSP in the group without ICC fits the assumption of a prevalence of about 10% [17], in contrast to much less chronic pain in the ICC cohort, reinforcing our hypothesis. Even when looking at the complete study population, our cohort seems to have significantly less cases of CPSP as one would expect from the literature [10].

In recent years, a primary VATS approach has been demonstrated to be the superior method for lobectomies when compared to traditional open surgery techniques for early-stage lung cancer. The increasing popularity of VATS has resulted in improved patient well-being and outcomes. According to the literature, the incidence of CPSP has been reduced from 47% in thoracotomy procedures, as reported by Bayman et al. [26], to 35% in VATS, as reported in Chen et al. [10]. In our cohort, we observed a CPSP rate from only up to 12.1% six months after surgery for the complete study cohort and up to 16.2% for patients without ICC. Further, the length of hospitalization and postoperative complications have been significantly reduced by the use of VATS [4,10,26].

Although improved perioperative pain management positively impacted patient outcome, an ideal, standardized, and universally agreed upon pain treatment regimen is lacking [6,8,11,12,14].

Post-surgical pain is still a major factor for patients’ well-being and quality of life, considering that 86% of all patients who undergo surgery experience postoperative pain [6,9,27].

Effective pain management comes hand-in-hand with early mobilization as well as the early opportunity for physiotherapy. This leads to fewer pulmonary side effects, such as atelectasis and the often subsequent pulmonary infections. Moreover, effective perioperative pain management may also decrease the appearance of CPSP [13,17,28]. It is noteworthy that satisfactory pain control is only possible with the permanent assessment and reassessment of pain levels and quality, especially due to pain being a subjective sensation, making the individual addressing of pain indispensable [20].

Opioids are traditionally an integral part in the management of acute and chronic pain due to their potent analgesic effects. However, their extensive and often unselective use has led to a growing health care issue from the rising rates of opioid misuse, addiction, and overdose deaths [15,18]. In the US, overdoses of opioids are responsible for more deaths than heroin and cocaine combined and is now the second leading cause of accidental deaths in the USA. Prescribed painkillers represent the second most common abused drug after marijuana in the USA. To face these problems, opioid-sparing analgesic approaches are needed, to prevent addiction and misuse in the beginning [15,16].

Opioids play a major role in postoperative pain control. In this setting, the use of opioids has been linked to some severe complications such as respiratory depression, sedation, constipation, and others, which negatively impact patient recovery and prolong hospitalization [16,18]. The prolonged use of opioids may also lead to paradox effects, such as opioid-induced hyperalgesia (OIH), where individuals react hypersensitively to painful stimuli, which can intensify pain rather than alleviate it. This may lead to even higher rates of opioid usage and higher dosing regimens, which, in turn, produces a higher tolerance in patients. Subsequent dose escalations cause a vicious cycle and further complicates postoperative pain control [29].

In response to these concerns, there has been an intensive effort in medical professions to explore alternative pain management strategies and reduce the reliance on opioids. Multimodal analgesia approaches, which combine opioids with other non-opioid analgesics and regional anesthesia techniques, have been shown to be effective in providing adequate pain relief while minimizing opioid consumption and the associated side effects [15,16,18]. Enhanced recovery after surgery (ERAS) protocols, which integrate a multimodal approach to perioperative care, have also gained popularity and have been associated with reduced opioid use, shorter hospital stays, and improved patient outcomes [5,18]. Nevertheless, these protocols also possess possible downsides. Epidural anesthesia may provide excellent pain relief perioperatively, with the advantage of precise dosing and dose titration to achieve the desired level of anesthesia, offering flexibility in pain management. Potential complications include postinterventional hypotension, headache, bladder disfunction, and rare but serious complications such as epidural hematoma, abscess, or possible damage to the spinal cord. The procedure itself carries risks, including accidental dural puncture and infection, which may necessitate further interventions and treatments. Epidural anesthesia often comes with technical difficulties due to anatomical variations and can be rather time-consuming, which might be an important factor in times of reduced operating room capacities due to understaffing. ICC represents a feasible and easy alternative with less severe side effects that also brings the possibility of pain control for a prolonged period of up to three days after surgery. As our study group has already shown, ICC placement does not significantly prolong operating time [7,30,31]. Moreover, in this follow-up study, the operating time was not prolonged (ICC: 153.16 vs. No ICC: 155.68 min, *p* = 0.681). One of the downsides of ICCs consists in the needed interdisciplinary postoperative management, which may lead to additional administrative work. Nevertheless, this can be successfully minimalized by the close co-operation between the surgical, anesthesiologic, and pain management specialties [7,13,14]. An ultrasound-guided erector spinae plane block (ESPB) or thoracic paravertebral block (PVB) might pose as further alternatives for regional pain management. A meta-analysis conducted by Fenta et al. showed superior results in terms of pain control during movement in the first 12 postoperative hours and less opioid consumption in PVB patients when compared to ESPB [32]. Even though ESPB also seems to show inferior results compared to epidural anesthesia, it still provides better pain control than intravenous opioids or SSINB alone [33,34,35] Although recent meta-analyses show the benefits of regional thoracic wall blocks, data on ICC with the continuous application of ropivacaine are still lacking and need further investigation [36,37].

Those who suffer from chronic pain also have a measurably worse quality of life. Their everyday life is influenced in many aspects. Chronic pain can lead to depression, sleep issues, and fatigue. It is linked to a reduced ability to work and loss of productivity. Besides the personal burden for each individual, chronic pain causes extensive economic burdens for society and health care systems [38].

For measuring health-related quality of life (HRQoL), the Eqol-5D (EQ-5D) is a widely used tool containing five comprehensive questions about mobility, self-care, ability to undertake usual activity, pain/discomfort, and anxiety/depression [39]. The PROGRESS trial [39] showed EQ-5D levels of 0.15, on average, for chronic pain patients, which are significantly lower than for hospitalized stroke patients with an EQ-5D of 0.31, with 0 being a score equal to death and 1.00 meaning good health [38]. As a comparison, general population norms in Norway for the EQ-5D are 0.82 [40]. The associated economic consequences of chronic pain have a rather high impact compared to other health conditions. Due to the fact that chronic pain can influence the ability to work, and also effects the productivity of the affected individuals, socio-economic costs may be multiplied [38]. CPSP with a prevalence of approximately 10%, and even higher rates after thoracic surgery, constitutes a severe public health issue. Considering an aging population in most countries and an increasing number of surgical treatments, especially for primary lung cancer, a constantly rising prevalence can be assumed [17].

Although the definition of CPSP varies, most of the literature agrees that the location and duration of pain serve as the criteria. Pain existing for a period longer than one month of what the affected tissue usually needs to heal in an operation-associated region is defined as the CPSP. On average, this period is about three to six months. Despite this rather long time frame for possible healing, CPSP accounts for about one-third of all chronic pain patients [17].

The potential risk factors for CPSP after thoracoscopic surgery are the duration of drainage, number of incisions, and inadequate pain control perioperatively [10]. The prolonged use of opioids and poorly controlled acute pain after surgery may contribute to the development of CPSP [16]. Although Chen et al. [10] reported female sex, beneath other factors, as a definite risk factor for CPSP, we did not find any differences in our results when stratified by gender.

When asked for the location of pain in the follow-up assessments, many patients complain about the area where the chest drainage was inserted. A targeted regional anesthesia seems to result in an obvious improvement of this special problem [6,8,31,41].

Our findings lead to the assumption that ICC placement has positive short- and long-term effects in pain management after thoracic surgery and reduces the prevalence of CPSP. These results demonstrate the superiority of using ICC in combination with SSINB, in comparison to SSINB alone, as an integral part in postoperative pain management.

### Limitations

This analysis was limited by the retrospective character and its small sample size. A prospectively designed analysis would likely yield more accurate and detailed data regarding the characteristics of pain and potential side effects associated with the use of ICCs. Additionally, our database contains no information on patients’ quality of life and return to daily activity.

## 5. Conclusions

This follow-up study is able to demonstrate the effectiveness of ICC placement in patients undergoing VATS and highlights the significant short- and long-term advantages of ICCs over SSINB alone. The findings of this study show that patients who received an ICC required a reduced quantity of opioids and less frequent administration of opioids, when compared to patients without an ICC. Further, patients with ICC expressed less pain in every follow-up consultation and experienced a significant lower risk of developing CPSP one year after surgery. These results highlight the potential benefits of ICC placement in VATS patients, not only in terms of improved pain management, but also in reducing the long-term risks associated with opioid use and the development of CPSP. Further research and larger prospective studies are warranted to validate these findings.

## Figures and Tables

**Table 1:** Patient demographics.

	ICC (*n* = 67)	No ICC (*n* = 74)	*p*-Value
Age [years], median (Mean)	65.00 (64.23)	65.00 (64.73)	0.766
Sex			0.615
- Female	34 (50.7)	41 (55.4)	
- Male	33 (49.3)	33 (44.6)	
LOS [days], median (Mean)	6.00 (6.99)	6.00 (7.43)	0.536
Side (%)			
- Left lung	20 (29.9)	29 (39.2)	
- Right lung	47 (70.1)	45 (60.8)	
Lobe (%)			0.909
- Lower	28 (41.8)	28 (37.8)	
- Upper	31 (46.3)	39 (52.7)	
- Middle	7 (10.4)	6 (8.1)	
- Multilobar	1 (1.5)	1 (1.4)	
Diabetes mellitus (%)	7 (10.4)	11 (14.9)	

## Data Availability

The data presented in this study are available upon request from the corresponding author. The data are not publicly available due to institutional privacy policies.
